# A Treatise on Asiatic Cholera

**Published:** 1885-08

**Authors:** 


A Treatise On Asiatic Cholera. *Edited and prepared by* Edmund Charles Wendt, m. d., *New York. New York:* William Wood & Co. 1885. *Chicago:* W. T. Keener, *96 Washington St.*

The publication of this treatise, at this time, is an opportune and valuable contribution to medical literature. It is especially valuable to American readers in presenting, in careful detail, a history of cholera since its first appearance in this country in 1832. Its course and progress are marked across the continent with the precision of the advance of a successful army, and its frequent repulses are justly referred to intelligent and persistent sanitary precautions. The history of the disease, as presented in this volume, constitutes the strongest possible plea for this mode of defence. The various theories of causation an given in chronological order, including the recent alleged discoveries of Koch as presented at the Berlin Cholera Conference in July, of last year, and the later researches of Maurin and Lange. The subject has been treated in all its aspects in a highly satisfactory manner, and all the mooted questions, relating to the cause, propagation, prevention, and treatment of the disease, have been discussed in a judicial spirit, as free as possible from editorial prejudice. The volume is well entitled to a place in medical libraries.

